# The prevalence and cost of medical student visiting rotations

**DOI:** 10.1186/s12909-016-0805-z

**Published:** 2016-11-14

**Authors:** Matthew Winterton, Jaimo Ahn, Joseph Bernstein

**Affiliations:** Department of Orthopaedic Surgery, University of Pennsylvania School of Medicine, 424 Stemmler Hall, Philadelphia, USA

**Keywords:** Visiting student rotation, Away rotation, Residency, Residency application, Medical school, Medical education

## Abstract

**Background:**

Performance on visiting rotations during the senior year of medical school is consistently cited by residency program directors as a critical factor in selecting residents. Nevertheless, the frequency with which visiting rotations are undertaken and the associated financial costs they impose have not been systematically examined.

**Method:**

Under the auspices of the Electronic Residency Application Service, a survey was sent in March 2015 to all U.S. applicants for residency programs in the 2014-15 academic year. Students were asked how many visiting rotations they performed; the estimated cost of performing each rotation; their perception of their educational value and primary motivation for performing them; and the Match outcome of their residency application.

**Results:**

The survey was completed by 2817 applicants, yielding a response rate of 11.3 %. 1898 applicants (67.4 %) performed visiting rotations: 647 applicants (30.0 %) performed one; 640 (22.7 %) performed two; 322 (11.4 %) performed three; and 289 (10.3 %) reported four or more. When accounting for potential response bias, the true prevalence of away rotators was estimated to be 58.7 % of all fourth-year medical students (95 % CI 54.0–63.4 %). The mean number of rotations for participating students was 2.1. Most students performed rotations equally as an audition for residency placement and for education, with some of the more competitive subspecialties reporting more of an audition experience. The mean estimated cost for performing a single rotation was $958. Thirty-six percent of applicants reported matching at an institution where they had rotated, either their home institution or one at which a visiting rotation was performed.

**Conclusions:**

Visiting rotations are prevalent, expensive, and only partly educational. As such, these rotations may impede optimal use of the senior year of medical school and limited student financial resources.

## Background

According to Ludmerer, the basic structure of medical school curricula was established in the 19^th^ century: “The first 2 years contained the pre-clinical disciplines….[and the] last 2 years provided instruction in the various clinical subjects” [1]. Within that broad partition, the initial 2 years were subdivided into the study of normal biology in the first year and diseases in the second; and within the latter half, the third year was devoted to “major” clerkships and the fourth year left open for electives.

Although the fourth year has been “relatively ignored in curricular reforms” [2], it has been implicitly modified by changes made to the other years’ content and structure. For one thing, at many schools, traditional third year clinical clerkships now start before the third academic year. (At our institution, clerkships begin in January of the second year.) And because the clerkships likewise end earlier, the fourth year is for many students an expanded 18 month-long segment, replete with opportunities for electives.

One such opportunity opened to students is the freedom to visit other medical schools, outside of the students’ home institution, often in conjunction with application to residency programs [3]. We have observed informally that visiting rotations have become increasingly popular. At our home institution, for example, over the years 2012 to 2015, there was a near doubling of the number of visiting rotations [Helene Weinberg, Registrar; personal communication]. Along those lines, in the 2014–2015 application cycle alone, 129,874 applications for visiting rotations were submitted by 13,273 applicants through the Visiting Student Application Service [Association of American Medical Colleges Visiting Student Application Service Database, as of 5/28/2015. Last updated 5/28/2015].

There is, of course, a good reason for the popularity of visiting rotations among students: namely, the popularity of visiting rotations among program directors who select residents. Surveys of program directors [4–9] demonstrate that performance on a visiting rotation is one of the most important factors in selecting candidates for interview [10], especially in some competitive specialties [8, 11].

Despite the apparent increasing importance of visiting rotations in residency placement, we have found no reports of the overall prevalence of these visiting rotations; and while there have been reports on their purported benefits [12], we have not found reports on the costs associated with performing these visiting electives. We address these questions here.

## Methods

Under the auspices of the Electronic Residency Application Service (ERAS®, the Association of American Medical Colleges service for residency applications) an anonymous web-based survey was sent in March 2015 to all U.S. applicants for allopathic residency programs in the 2014-15 academic year. This survey asked students how many visiting rotations they performed; the estimated dollar cost of performing each visiting rotation; their perception of the educational value of these rotations; and the match outcome of their residency application.

Pearson’s chi-square test was used to examine the goodness-of-fit of the distribution of respondents disclosing their primary specialty in which they applied and the actual distribution of applicants within each specialty nationally, as gathered by the National Resident Matching Program® [13]. Significance level was set at *P* = 0.05. Statistical analysis was performed using Microsoft Excel (Microsoft Corporation, Redmond, WA).

To evaluate potential non-response bias, we distributed a second survey 2 weeks later to those students who did not originally respond asking how many visiting rotations they performed. Using a variable response propensity model [14] to correct for survey non-response, we estimated the proportion of students who did not perform visiting rotations.

## Results

**Fig. 1:**
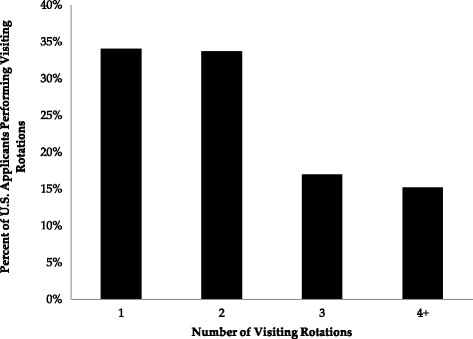
Distribution of visiting rotations performed among U.S. applicants

The survey was completed by 2817 applicants, yielding a response rate of 11.3 % (Table 1). We found that 1898 applicants (67.4 %) reported performing visiting rotations: 647 applicants (30.0 %) performed one; 640 (22.7 %) performed two; 322 applicants (11.4 %) performed three; and 289 (10.3 %) reported performing four or more visiting rotations (Fig. 1). The mean number of rotations for participating students was 2.1. The second survey evaluating non-response bias was completed by 1681 applicants, yielding a response rate of 7.6 %. 1006 applicants (59.8 %) reported performing visiting rotations: 365 (21.7 %) performed one; 330 (19.6 %) performed two; 158 (9.4 %) performed three; and 153 (9.1 %) reported four or more.

**Table 1:** Survey Responses

	Number	Fraction of *n* that did VR	Mean number of VR^a^	% matching at places where auditioned^b^
Anesthesiology	118	60.2 %	1.7	37 %
Dermatology	45	88.9 %	2.3	47 %
Emergency Medicine	148	93.2 %	2.0	43 %
Family Medicine	148	58.8 %	2.4	47 %
Internal Medicine	255	43.5 %	2.0	29 %
Neurological Surgery	27	100.0 %	2.4	30 %
Neurology	36	50.0 %	2.2	26 %
Obstetrics and Gynecology	115	61.7 %	2.0	33 %
Ophthalmology	37	73.0 %	1.9	32 %
Orthopaedic Surgery	80	98.8 %	2.4	56 %
OTHER/Specialty not reported	1277	70.6 %	2.1	37 %
Otolaryngology	42	92.9 %	1.8	36 %
Pediatrics	203	55.7 %	2.3	29 %
PM&R	28	71.4 %	2.1	29 %
Plastic Surgery	18	100.0 %	2.4	44 %
Psychiatry	65	47.7 %	1.8	32 %
Radiation Oncology	13	84.6 %	2.4	46 %
Radiology-Diagnostic	58	48.3 %	2.0	25 %
Surgery	104	65.4 %	2.0	25 %
Total	2817	67.4 %	2.2	36 %

Abbreviations: *VR* visiting rotation, *PM&R* physical medicine and rehabilitation

^a^Among those applicants who performed a VR

^b^VR location or home

Using a variable response propensity model [14], the proportion of all students that performed zero visiting rotations, including early respondents, late respondents, and non-respondents, was 41.3 % (95 % CI 36.6–46.0 %).

There were 1540 respondents (54.7 %) who reported the specialty in which they primarily applied (Table 1). The data demonstrate an excellent cross-section of the distribution of applicants nationally (*P* > 0.999), indicating that our sample was not overly influenced by any particular specialty. Visiting electives were most prevalent among neurological surgery and plastic surgery applicants, with 100 % of survey respondents performing one or more visiting rotations. Over 90 % of emergency medicine, orthopaedic surgery, and otolaryngology applicants each performed at least one visiting rotation. Visiting rotations were least prevalent among internal medicine (43.5 %), psychiatry (47.7 %), and diagnostic radiology (48.3 %) applicants.

Among those who performed visiting rotations, family medicine, neurological surgery, orthopaedic surgery, plastic surgery, and radiation oncology applicants each performed the highest average number of visiting rotations (2.4; Table 1). Anesthesiology applicants performed the least average number of visiting rotations (1.7).

Most visiting rotations (45 %) were deemed by respondents to have value for both learning and job hunting (Table 2). Very few visiting rotations were described as having an exclusively audition (9 %) or educational (7 %) purpose. Within the competitive specialties of orthopaedic surgery, otolaryngology, and plastic surgery, applicants’ description of their visiting rotations skewed toward a mostly audition experience than an educational one.

**Table 2:** Rationale for performing visiting rotations

Specialty	Purely audition	Mostly audition	Equal audition and education	Mostly education	Purely education
Anesthesiology	8 %	25 %	45 %	15 %	7 %
Dermatology	16 %	21 %	41 %	17 %	6 %
Emergency Medicine	9 %	24 %	50 %	13 %	4 %
Family Medicine	15 %	21 %	43 %	12 %	9 %
Internal Medicine	2 %	17 %	47 %	21 %	12 %
Neurological Surgery	7 %	31 %	48 %	10 %	5 %
Neurology	3 %	17 %	64 %	14 %	3 %
OBGYN	8 %	28 %	46 %	12 %	6 %
Ophthalmology	4 %	27 %	48 %	12 %	10 %
Orthopaedic Surgery	13 %	46 %	33 %	7 %	2 %
Otolaryngology	5 %	55 %	31 %	8 %	2 %
Pediatrics	9 %	11 %	52 %	18 %	11 %
PM&R	5 %	36 %	41 %	15 %	3 %
Plastic Surgery	14 %	43 %	39 %	2 %	2 %
Psychiatry	2 %	18 %	44 %	16 %	20 %
Radiation Oncology	8 %	38 %	46 %	8 %	0 %
Radiology-Diagnostic	6 %	30 %	43 %	15 %	6 %
Surgery	10 %	27 %	45 %	15 %	4 %
Total	9 %	26 %	45 %	14 %	7 %

Abbreviations: *OBGYN* obstetrics and gynecology, *PM&R* physical medicine and rehabilitation. Data are shown as percentages of the total number of visiting rotations within each specialty

Overall, among all survey respondents, 36 % of applicants reported matching at an institution at which they had been seen previously, either their home institution or one at which a visiting rotation was performed (Table 1). Orthopaedic surgery applicants were the most likely to match either at their home institution or at a visiting rotation (56 %), whereas diagnostic radiology and general surgery applicants were the least likely (25 %).

When examining only those survey respondents performing visiting rotations, 42 % matched at an institution where they rotated, either their home institution or at an institution where a visiting rotation was performed. Within the specialties, psychiatry applicants who performed at least one visiting rotation matched most frequently at an institution where they had been seen previously (58 %), which was closely followed by orthopaedic surgery (57 %), radiation oncology (55 %), and family medicine (54 %) applicants. The specialties matching least frequently at an institution where they had been seen previously were general surgery (28 %) and neurology (28 %).

**Fig. 2:**
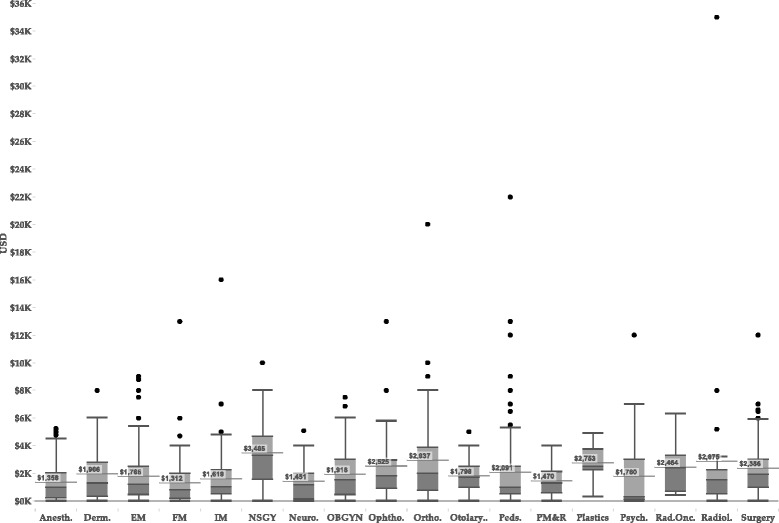
Tukey boxplots of dollar costs of performing total away rotations by specialty. Mean dollar costs are shown as a horizontal line with dollar value

The mean estimated dollar cost for performing a single visiting rotation was $958. As expected for specialties in which higher numbers of visiting rotations were performed, neurological surgery applicants reported the highest mean total expenses for visiting rotations performed (Fig. 2; $3,465), followed by orthopaedic surgery ($2,937) and diagnostic radiology ($2,875). The least total expenditures were from family medicine ($1,312) and anesthesiology ($1,358).

## Discussion

Visiting rotations are prevalent, with a corrected rate of 58.7 % of survey respondents reporting performing one or more. The high prevalence of visiting rotations may be a good thing. To start, students can learn, reconnoiter and audition. Further, by visiting, applicants can demonstrate their sincere interest in the visited institution. Visiting rotations also may allow the host institution to examine what might be termed the applicants’ “affective strengths and weakness” — elements of their personality, such as extraversion, agreeableness, openness, conscientiousness and absence of neuroticism [15]. These features may be otherwise obscure on transcripts and recommendation letters, and nonetheless may be important [16].

Similarly, by noting the applicant’s decision to visit, the institution may reasonably (and more accurately) infer interest in the program., Asking residents about their commitment is of course forbidden. As noted by the AAMC [17], “It is a violation of the Match Participation Agreement for programs to request that applicants reveal their ranking…” Nevertheless, programs seem to be keenly interested in applicants level of commitment [18] and noting the performance of a visiting rotation is a licit means of gaining that information.

Despite the benefits of visiting rotations, there are problems to consider. For one thing, these rotations have a high dollar cost. In addition, visiting rotations have a high opportunity cost [9, 19, 20] as well: time spent auditioning may be time lost from more educational endeavors. Additionally, a system of resident selection that favors those who perform visiting rotations confers a perhaps unfair advantage on wealthier students who can afford to visit multiple sites, or on students whose school schedule (i.e., ending clerkships in December, not June) is more conducive to extensive travel.

It is likely that in some fields applicants may view the visiting rotation as a de facto requirement: as noted, in competitive specialties such as plastic surgery, neurological surgery, orthopaedic surgery, emergency medicine, and otolaryngology, nearly all applicants performed visiting rotations, and in those fields there was a greater tendency to report the visiting rotation experience as having a greater audition component. At competitive programs, selection committees may believe that only those students who have been seen –either as part of a visiting rotation or as a home institution course—are eligible for further consideration.

Performing the mean number of visiting rotations can be expensive. Whereas the average applicant spent approximately $2,000 on visiting rotations, many applicants spent in excess of $5,000 and $10,000 on these visiting rotations. Moreover, this estimate does not include costs the home institution may impose, to say nothing of the costs, financial and other, that are incurred attempting to arrange rotations that are not ultimately undertaken.

We acknowledge limitations to our study. While the number of respondents was large and well distributed, there very well may be selection bias, in which those students who performed a visiting rotation would be more likely to participate. In an effort to mitigate such non-response bias, and to estimate the true prevalence of away rotations among fourth-year medical students, we distributed a second survey to those students who did not response the first time. As expected, this cohort of respondents performed fewer visiting rotations than the original respondents. Using a variable response propensity model, we deduced that the proportion of all students that performed zero visiting rotations, including early respondents, late respondents, and non-respondents, was 41.3 % (95 % CI 36.6–46.0 %), indicating that the true proportion of all fourth-year medical students performing a visiting rotation was 58.7 % (95 % CI 54.0–63.4 %). With a slightly higher percentage of students reporting performing visiting rotations, the first survey responses were slightly biased toward students performing visiting rotations.

Also, because no demographic information was collected, we cannot be sure that the sample we have reflects the student population at large. It would have been more informative to collect information on the geographic region of the students’ schools; the size and organization (public vs private) of the medical school; and the presence or absence of a residency program in the desired field at the individual respondent’s school. It also would have been more informative to collect information on the duration of the rotation, as clearly a 4 week rotation imposes a larger opportunity cost (to say nothing of financial cost, perhaps) than one only 2 weeks in length.

The rating system we used for the educational/audition value of the rotation probably suffers from a central tendency bias: namely, the predisposition of many respondents, in general, to avoid the extremes of the rating scale and thereby (incorrectly) favor scores near the midpoint. Even with that potential limitation, comparisons within specialties may be interesting nonetheless, as the bias would apply equally to all. It must be noted, further, that contrasting the educational versus audition value of the rotation is not the only dimension on which fair comparisons may be made. For example, rotations may be taken to compensate for a deficiency of the home institutions’ offerings, and limiting the analysis to this single vector may omit some salient costs and benefits.

Last, the cost estimates are just that: estimates. It is certainly possible that individual respondents may have distinct ideas as to what “counts” as a rotation-related expense. For instance, a student may consider only those food costs above and beyond that which would have been spent at home (restaurants and cafeterias versus grocery stores and home-cooked meals) whereas some may have considered the entire food expense.

More than that, these estimates are given many months after the fact, a feature that introduces a bias (or imprecision, at least) owing to the reliance on memory.

Despite these limitations, this study may help inform a needed discussion. Currently, only about 10 % of applicants perform 4 or more visiting rotations. Nonetheless, it is easy to imagine a burgeoning “arms race” [21] in which applicants routinely devote all available time to auditioning, to the exclusion of other scholarly pursuits. And because it is not reasonable to ask individual schools to unilaterally disarm (that is, limit their own students’ travels, and thereby place them at a competitive disadvantage), discussion within our community for consensus self-regulation is imperative.

## Conclusions

Visiting rotations are prevalent, expensive, and only partly educational. As such, these rotations may impede optimal use of the senior year of medical school and limited student financial resources.

## Abbreviation

*ERAS*: Electronic residency application system
